# Sociodemographic and biological determinants of insulin initiation in type 2 diabetes: a cohort study using routinely collected primary care data

**DOI:** 10.3399/BJGP.2024.0693

**Published:** 2025-10-21

**Authors:** Sukainah A Alfaraj, Rimke C Vos, Marco Spruit, Rolf HH Groenwold, Dennis O Mook-Kanamori

**Affiliations:** 1 Department of Public Health and Primary Care/Health Campus the Hague, Leiden University Medical Center (LUMC), Leiden, the Netherlands; 2 Leiden Institute of Advanced Computer Science, Leiden University, Leiden, the Netherlands; 3 Department of Biomedical Data Sciences, LUMC, Leiden, the Netherlands; 4 Department of Clinical Epidemiology, LUMC, Leiden, the Netherlands

**Keywords:** healthcare disparities, insulin initiation, primary health care, sociodemographic factors, type 2 diabetes

## Abstract

**Background:**

Timely initiation of insulin is critical to prevent long-term complications associated with poor glycaemic control. A better understanding of the factors influencing insulin initiation is essential to guide person-centred treatment and reduce disparities.

**Aim:**

To examine factors associated with insulin initiation within 5 years after starting metformin in adults with type 2 diabetes mellitus (T2DM).

**Design and setting:**

Cohort study using ELAN primary care data linked to Statistics Netherlands registry data.

**Method:**

Adults aged 40–79 years with T2DM who initiated metformin between 2007 and 2023 were included. Fine and Gray competing risk analysis was used to assess time to insulin initiation while accounting for death. Covariates measured within 6 months of metformin initiation included age, sex, smoking status, body mass index (BMI), systolic blood pressure, country of origin, household income, household support, glycosylated haemoglobin (HbA^1c^), estimated glomerular filtration rate (eGFR), total cholesterol, and high-density lipoprotein (HDL) cholesterol.

**Results:**

Among 24 360 individuals with T2DM on metformin, 2326 (10%) initiated insulin within 5 years. Compared with individuals of Dutch origin, insulin initiation was less likely among people with Surinamese (subdistribution hazard ratio [SHR] 0.62, 95% confidence interval [CI] = 0.45 to 0.79), Turkish (SHR 0.61, 95% CI = 0.30 to 0.91), or other backgrounds other than Dutch (SHR 0.76, 95% CI = 0.61 to 0.90). Higher HbA1c increased the likelihood of insulin initiation (SHR 1.54 per 13 mmol/mol, 95% CI = 1.51 to 1.57). Factors associated with a lower probability of insulin initiation included higher household income (SHR 0.94 per 28% increase, 95% CI = 0.89 to 0.98), overweight or obesity versus having a healthy BMI, older age, higher eGFR, higher HDL, and later calendar year.

**Conclusion:**

Besides biological factors, backgrounds other than Dutch and higher household income are linked to a lower probability of initiating insulin therapy.

## How this fits in

Insulin initiation in type 2 diabetes mellitus remains challenging despite clear clinical guidelines. Although previous studies have identified biological factors influencing insulin use, less is known about the impact of migration background and socioeconomic status in routine primary care. This study demonstrates that social and cultural factors play a role in insulin initiation, beyond biological markers. Addressing these differences is important to support equitable diabetes management in a diverse patient population.

## Introduction

Type 2 diabetes mellitus (T2DM) is a chronic metabolic disorder characterised by insulin resistance and relative insulin deficiency.^1^ As the disease progresses, managing T2DM often requires a comprehensive approach that includes lifestyle changes, oral hypoglycaemic agents, and potentially daily insulin therapy.^2^ Timely intensification of medication is crucial to maintain glycaemic control and prevent or delay complications.^3,4^

The American Diabetes Association and the European Association for the Study of Diabetes have established a joint consensus algorithm to guide treatment decisions, emphasising individualised care that considers both healthcare providers’ and patients’ perspectives.^5^ This focus on person-centred treatment is especially important for insulin prescription.^6^ Although insulin is highly effective in lowering blood glucose levels, it is often viewed as a last resort when oral agents fail to provide adequate control.^7,8^ Barriers to insulin initiation include concerns about hypoglycaemia, patient adherence, limited consultation time, and availability of resources such as nurses and health educators.^9–12^ However, prolonged inadequate glycaemic control can lead to advanced beta-cell failure.^13^ Studies using UK modelling data and US real-world data found that delaying insulin use in the presence of poor glycaemic control was associated with a reduction in quality-adjusted life expectancy, a higher incidence of complications, and a lower probability of attaining good glycaemic control with insulin.^14,15^ This highlights the need for a better understanding of when to use insulin to avoid prolonged periods of hyperglycaemia. The decision to initiate insulin is complex and differs based on the unique circumstances of each patient and the perceptions of healthcare providers.^16^

Previous studies have highlighted various factors influencing insulin initiation in individuals with T2DM. Socioeconomic status (SES) has been associated with insulin use, with individuals from lower-income backgrounds more likely to require insulin therapy.^17^ Ethnic disparities have also been observed; some ethnic minority groups initiate oral treatment earlier but experience delays in advancing to insulin.^18^ A large primary care study from South Africa identified additional barriers such as stigma, fear of injections, and limitations in healthcare resources.^19^ The Look AHEAD trial, a large multicentre randomised study, investigated predictors of insulin initiation using Cox models adjusted for time-varying glycosylated haemoglobin (HbA1c) and multiple clinical and sociodemographic variables. It was found that younger age, a larger number of diabetes complications, and White ethnic group were associated with a higher likelihood of insulin initiation, whereas SES was not associated with insulin initiation.^20^ Although this study provided valuable insight, it was conducted within a structured trial setting. The current study complements and expands on this work by using real-world, routinely collected data from primary care to assess the influence of a broad set of clinical, sociodemographic, and biological factors on insulin initiation in people with T2DM within 5 years after metformin initiation. This helps address a key gap in understanding how these factors influence treatment escalation in everyday practice.

## Method

Data from the Extramural LUMC Academic Network (ELAN) dynamic registry was used, which is a database collected from the electronic health records of the GPs in South Holland, the Netherlands. The ELAN database contains comprehensive medical information for each registered patient. Medical information includes diagnosed diseases, prescribed medications, and an array of laboratory measurements. Further description of ELAN data is provided elsewhere.^21^ Data were pseudonymised to prevent the identification of individual patients. This study was assessed by the Medical Ethics Committee of Leiden Den Haag and Delft (METC-LDD). In accordance with institutional guidelines and the nature of the study, which involved the analysis of pseudonymised routinely collected health data, a non-WMO (Wet medisch-wetenschappelijk onderzoek — the Dutch Medical Research Involving Human Subjects Act) statement was granted under METC number N22.006, with a waiver to obtain informed consent.

**Figure 1.**
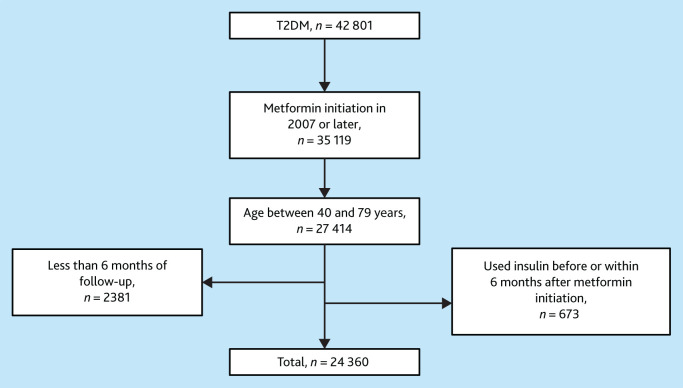
Flow chart illustrating the selection process of the study population and exclusion criteria for the analysis of factors influencing insulin initiation in individuals with T2DM treated with metformin. T2DM = type 2 diabetes mellitus.

The study population included individuals with T2DM, aged 40–79 years, who started metformin treatment between January 2007 and July 2023 (Figure 1). T2DM diagnoses were identified using the International Classification of Care code T90.02. Metformin users were identified through prescription data using the Anatomical Therapeutic Chemical (ATC) code A10BA02, and their initial metformin prescription date were traced back to August 2000. ELAN data collection started in January 2007. To ensure data accuracy and to reduce the amount of missing information, metformin users from the year 2007 onwards only were included. Insulin users were identified using the ATC code A10A and prescription date to determine their first insulin prescription. The beginning of the follow-up period (that is, baseline) was designated as 6 months following the initiation of metformin treatment. The rationale behind this 6-month interval is to afford patients an adequate duration for metformin to lower glucose and have reliable measures for physiological parameters. Thus, patients who had an insulin prescription before starting metformin or their insulin initiation coincided with the start of metformin treatment were excluded (*n* = 673) from the study to focus on newly diagnosed patients with T2DM who had not previously received insulin treatment and exclude other possible types of diabetes that could be initially misdiagnosed as T2DM. Additionally, patients were excluded who did not have any follow-up after the start date of metformin or had their last contact with their GP within this 6-month period (*n* = 2381).

### Outcome and predictors

The primary outcome was the time to initiation of insulin therapy within a 5-year follow-up period. The selection of potential predictors for insulin initiation in this study was informed by their established associations with diabetes management and patient outcomes; each was considered independent of one another. Age is essential in determining treatment plans, as the target HbA1c level and, consequently, the intensification of antidiabetic medication depends on the patient’s age.^22,23^ Similarly, sex differences in metabolic responses and complications may influence treatment decisions, making it an important predictor.^24^ Smoking status has been linked to worse glycaemic control and an elevated risk of diabetes-related complications.^25^ Body mass index (BMI) is a significant risk factor, with higher levels being related to greater insulin resistance.^26^ Hypertension, dyslipidaemia, and chronic kidney disease are common comorbidities in diabetes that correlate with cardiovascular risk, which is a key determining factor in the choice and intensification of antidiabetic medications.^22^ In this study, systolic blood pressure, estimated glomerular filtration rate (eGFR), high-density lipoprotein (HDL), and total cholesterol measurements were used to assess these comorbidities and their impact on insulin initiation. The measurement closest to the index time (6 months after the initiation of metformin) was used for each laboratory test.

Additional information was obtained from Statistics Netherlands registry data, including household income and support, ethnic background, and death registration. Disposable household income refers to the remaining income available to a household each year after taxes and other deductions, adjusted for the household’s size and composition. This household income serves as an indicator of SES.^27^ Data from Statistics Netherlands provide information on the distribution of disposable household income in relation to the general population, expressed as percentiles (0% to 100%). SES was categorised based on national quintiles of annual income (€16 000, €21 100, €26 800, and €34 700). Household support was assessed based on whether individuals lived alone or had support systems through other adults in the household.

For the classification of migration background, individuals were categorised based on their country of origin, using a combination of criteria that include the place of birth of the individual and their parents. The country of origin was divided into Dutch, Surinamese, Indonesian, Moroccan, Turkish, and other ethnic group. Death registration includes the date of death and the cause of death, coded according to International Classification of Diseases, 10th Revision codes.

### Statistical analysis

Fine and Gray survival analysis was used to assess the subdistribution hazard rate of insulin initiation while considering death as a competing risk. Failing to account for competing risks may lead to overestimation of the event probability. This method allowed the authors to account for the competing nature of the event, providing more accurate estimates of risks in the presence of a competing event. Follow-up time for each individual was calculated from 6 months after the start of metformin treatment to either the initiation of insulin therapy, death, or the end of the 5-year follow-up period, whichever occurred first. All continuous predictors were standardised by subtracting the mean and dividing by the standard deviation before inclusion in the regression model. This approach allows for comparability of effect sizes across numeric variables. Effect estimates are shown per one standard deviation increase.

The proportional subdistribution hazards assumption was assessed by testing for time-varying effects of covariates and found no evidence of violation. The relationship between BMI and insulin initiation was found to be non-linear; therefore, BMI was included in the model as a categorical variable: healthy weight (18–24.9 kg/m^2^), overweight (25–29.5 kg/m^2^), and obese (≥30 kg/m^2^).

Cumulative incidence functions (CIFs) were plotted to examine insulin initiation over the 5-year follow-up period, stratified by country of origin and SES.

There were approximately 5% missing data. In the case of missing data, numerical variables (biomarkers and income) were assumed to be missing at random, and binary variables, such as smoking status, were considered absent when data were missing. Smoking status at index date was categorised as current smoker or non-smoker. Individuals recorded as ex-smokers at the index date were grouped with non-smokers. Multiple Imputation by Chained Equations (MICE) was used to generate five imputed datasets for the missing laboratory measurement.^28^ MICE was performed including all variables in the final model, in addition to the outcome (insulin initiation), and the competing event (death) to ensure robustness of the imputed datasets. The results were pooled using Rubin’s rules.^29^ R Statistical Computing (version 4.2.1) was used for data preprocessing and analysis on the secure data infrastructure of Statistics Netherlands.^21^

## Results

In total, 24 360 individuals with T2DM who newly started treatment with metformin between the age of 40 and 79 years were identified. Within 5 years of the follow-up period, 2326 (10%) individuals started using insulin and 1025 (4%) patients died. The median duration of T2DM at the time of metformin initiation was 2 months (interquartile range [IQR] 0–7) for future insulin users, compared with 0 months (IQR 0–3) for future non-insulin users.

Table 1 shows the characteristics of study participants based on baseline measurement, stratified by insulin initiation within 5 years. Insulin initiators exhibited higher mean levels of HbA1c (61 versus 52 mmol/mol) compared with the non-insulin group. The mean household income was slightly lower in the insulin-initiated group compared with the non-insulin group. A higher percentage of Dutch (71% versus 67%), smokers (30% versus 27%), healthy BMI (16% versus 14%), and a lower percentage of overweight (38% versus 40%) was observed in the insulin user group compared with the non-insulin user group.

**Table 1.** Characteristics of patients with type 2 diabetes mellitus who were metformin users, stratified by insulin use within 5 years of the start of metformin

Characteristic^a^	Total (*N* = 24 360)	Insulin (*n* = 2326)	Non-insulin (*n* = 22 034)
**Age, years, mean (SD)**	59.9 (10)	59.4 (10)	60.0 (10)
**Calendar year, mean (SD)**	2012 (5)	2010 (3)	2012 (5
**Sex**			
Male	13 673	1304 (56)	12 369 (56)
Female	10 687	1022 (44)	9665 (44)
**Smoking**			
Yes	6748	700 (30)	6048 (27)
No	17 612	1626 (70)	15 986 (73)
**Living alone**			
Yes	6302	663 (29)	5639 (26)
No	18 058	1663 (71)	16 395 (74)
**BMI, mean (SD)**	30.2 (5.40)	30.2 (5.77)	30.2 (5.40)
Healthy (18–24.9 kg/m^2^)	3544	383 (16)	3161 (14)
Overweight (25–30 kg/m^2^)	9692	877 (38)	8815 (40)
Obese (>30 kg/m^2^ **)**	11 124	1066 (46)	10 058 (46)
**Income (mean percentile), mean (SD)**	51.1 (28)	49.1 (28)	51.3 (28)
**SES quintile**			
1st	4702	498 (21)	4204 (19)
2nd	4944	490 (21)	4454 (20)
3rd	4796	456 (20)	4340 (20)
4th	5000	466 (20)	4534 (21)
5th	4918	416 (18)	4502 (20)
**Country of origin**			
Dutch	16 493	1648 (71)	14 845 (67)
Surinamese	1983	164 (7)	1819 (8)
Indonesians	1198	110 (5)	1088 (5)
Moroccan	921	94 (4)	827 (4)
Turkish	689	55 (2)	634 (3)
Other ethnic group	3076	255 (11)	2821 (13)
**HbA1c (mmol/mol), mean (SD)**	53 (13)	61 (17)	52 (12)
**Systolic blood pressure (mmHg), mean (SD)**	137 (18)	138 (19)	137 (18)
**Total cholesterol (mmol/L), mean (SD)**	4.8 (1.2)	4.9 (1.2)	4.8 (1.2)
**HDL (mmol/L), mean (SD)**	1.2 (0.3)	1.17 (0.3)	1.20 (0.3)
**Triglyceride (mmol/L), mean (SD)**	1.9 (1.13)	2.08 (1.3)	1.89 (1.1)
**eGFR (mL/min/1.73m** ^**2**^ **), mean (SD)**	81.0 (22)	80.7 (25)	81.0 (22)

^a^Values are *n* (%) unless otherwise indicated. BMI = body mass index. eGFR = estimated glomerular filtration rate. HbA1c = glycosylated haemoglobin. HDL = high-density lipoprotein. SD = standard deviation. SES = socioeconomic status.

Table 2 shows the relationship between patient characteristics and time to insulin. Elevated baseline HbA1c levels increased the probability of insulin initiation (subdistribution hazard ratio [SHR] 1.54 per 13 mmol/mol, 95% confidence interval [CI] = 1.51 to 1.57). An older age was associated with a lower probability of insulin initiation (SHR 0.87 per 10 years, 95% CI = 0.82 to 0.92). Surinamese (SHR 0.62, 95% CI = 0.45 to 0.79) and Turkish (SHR 0.61, 95% CI = 0.30 to 0.91) individuals had a lower probability of receiving insulin when compared with their Dutch counterparts. A higher household income (SHR 0.94 per 28% increase, 95% CI = 0.89 to 0.98) and a lower HDL-cholesterol (SHR 0.94 per 0.33 mmol/L, 95% CI = 0.89 to 0.99) were associated with a lower probability of insulin initiation. The probability of insulin initiation was lower in people who were overweight (SHR 0.84, 95% CI = 0.70 to 0.98) and those who were obese (SHR 0.85, 95% CI = 0.70 to 0.99) compared with people with a healthy BMI. Later calendar year of starting metformin was associated with a lower likelihood of insulin initiation (SHR 0.68 per 5 years, 95% CI = 0.62 to 0.73).

Sex, household support, smoking, systolic blood pressure, and total cholesterol were not found to be associated with insulin initiation. Fine and Gray regression analysis for the competing event (death) is provided in Supplementary Table S1. Non-standardised Fine and Gray regression analyses for the time to event (insulin initiation) and the competing event (death) are presented in Supplementary Tables S2 and S3, respectively.

**Table 2.** Relationship between characteristics of patients with type 2 diabetes mellitus who were metformin users and time to insulin initiation

Characteristic	SHR (95% CI)^a^	*P*-value
Female (reference)	1	0.075
Male	0.92 (0.82 to 1.01)	
Healthy weight (18–24.9 kg/m^2^, reference)	1	
Overweight (25–30 kg/m^2^)	0.84 (0.70 to 0.98)	0.013
Obesity (>30 kg/m^2^)	0.85 (0.70 to 0.99)	0.019
Dutch (reference)	1	
Surinamese	0.62 (0.45 to 0.79)	<0.001
Indonesian	0.84 (0.64 to 1.04)	0.080
Moroccan	0.84 (0.62 to 1.07)	0.142
Turkish	0.61 (0.30 to 0.91)	0.001
Other ethnic group	0.76 (0.61 to 0.90)	<0.001
Non-smoker (reference)	1	0.244
Smoker	1.06 (0.96 to 1.15)	
Living with adult (reference)	1	0.124
Living alone	1.08 (0.98 to 1.18)	
Age (per 10 years)^b^	0.87 (0.82 to 0.92)	<0.001
Calendar year (per 5 years)^b^	0.68 (0.62 to 0.73)	<0.001
Household income (per 28%)^b^	0.94 (0.89 to 0.98)	0.004
HbA1c (per 13 mmol/mol)^b^	1.54 (1.51 to 1.57)	<0.001
Systolic blood pressure (per 18 mmHg)^b^	0.98 (0.94 to 1.03)	0.489
Total cholesterol (per 1.2 mmol/L)^b^	0.97 (0.92 to 1.01)	0.130
HDL (per 0.33 mmol/L)^b^	0.94 (0.89 to 0.99)	0.019
eGFR (per 22 ml/min/1.73m^2^)^b^	0.91 (0.86 to 0.96)	<0.001

^a^SHRs are based on Fine and Gray competing risk regression analysis. ^b^Standardised variable; results are presented per 1 standard deviation increase. eGFR = estimated glomerular filtration rate. HbA1c = glycosylated haemoglobin. HDL = high-density lipoprotein. SHR = subdistribution hazard ratio.

**Figure 2.**
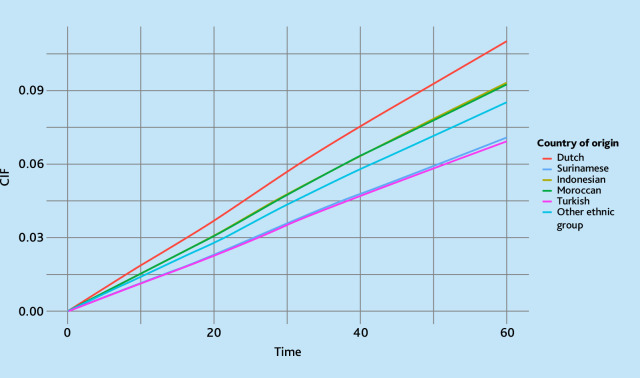
Cumulative incidence function (CIF) curves showing the probability of insulin initiation over time (in months), stratified by country of origin. Time is in months, starting 6 months after initiating metformin.

**Figure 3.**
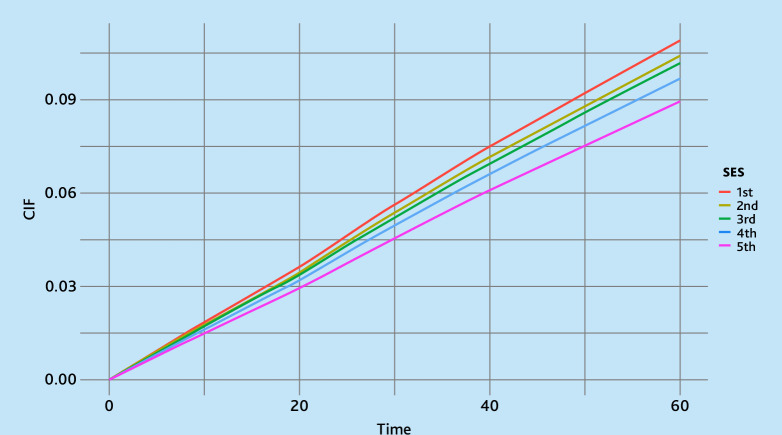
Cumulative incidence function (CIF) curves showing the probability of insulin initiation over time (in months), stratified by socioeconomic status (SES) based on disposable household income (1st is the lowest and 5th is the highest). Time is in months, starting 6 months after initiating metformin.

The CIF plots reveal disparities in time to insulin initiation across different countries of origin and SES groups (Figures 2 and 3). Insulin therapy was initiated earlier among individuals of Dutch origin compared with those with a migration background. Similarly, individuals in the lowest SES group showed a higher cumulative incidence, suggesting earlier initiation of insulin therapy than those in higher SES groups.

## Discussion

### Summary

The results of this study highlight a complex relationship between sociocultural and biological factors influencing the initiation of insulin therapy. The study found that some individuals with backgrounds other than Dutch, such as those with Surinamese and Turkish backgrounds, are less likely to receive insulin treatment within 5 years after initiating metformin. Furthermore, as anticipated, higher HbA1c levels at 6 months after metformin initiation and lower eGFR are significantly associated with an increased likelihood of starting insulin therapy. Individuals with lower income were more likely to receive insulin, which may reflect differences in disease progression or healthcare access. These findings add to existing literature by demonstrating these associations in a real-world primary care setting. Additionally, the findings highlight the need to better understand how ethnic group and SES influence treatment decisions in clinical practice.

### Strengths and limitations

The strength of this study lies in its utilisation of real-world data obtained from primary healthcare settings, providing insights that can better reflect routine clinical practices. Incorporating socioeconomic factors, such as country of origin and income, adds depth to the analysis by recognising elements beyond biological factors that may influence insulin prescription decisions. This comprehensive approach acknowledges the multifaceted nature of healthcare decision making.

The main limitation of this study is the inability to exclude other diabetes subtypes that necessitate early insulin prescription. Certain diabetes subtypes, specifically late autoimmune diabetes in adults (LADA), are often misdiagnosed as T2DM, present in people with normal BMI, and may require insulin therapy sooner than in people with T2DM.^30,31^ In this study there were challenges in identifying individuals with possible diagnoses of LADA owing to the absence of specific coding in the dataset in which they are usually classified as individuals with T2DM. Recognising and distinguishing these subtypes is crucial, as their unique clinical characteristics may influence both the timing and necessity of insulin prescription. The observed inverse relationship between a higher BMI and a reduced likelihood of insulin prescription revealed a weight-related consideration in clinical decision making.^32,33^ Although the well-documented phenomenon of insulin-induced weight gain offers a plausible explanation for this association, it is essential to acknowledge that lower BMI may also be indicative of LADA that necessitates earlier insulin initiation.^34^

Specific ethnic background was not available in the current dataset; therefore, country of origin was used as a proxy for ethnic group. Although this approach provides some insight into cultural and demographic differences, it may not fully capture the complexity of ethnic background. Additionally, the study considered one measurement for each covariate at index time, even though many of them are dynamically changing over time, such as HbA1c levels. Moreover, SGLT2 (sodium glucose co-transporter 2) inhibitors and GLP-1 (glucagon-like peptide-1) receptor agonists have recently begun receiving reimbursement in the Netherlands, which has led to their increased use in clinical practice. Thus, the use of SGLT2 inhibitors and GLP-1 receptor agonists was not accounted for in this study.

This study was observational in nature and aimed to explore factors associated with the initiation of insulin therapy. The study was not designed to establish causality, and the findings should be interpreted with caution as observational associations. Although the study adjusted for a range of relevant variables, some factors — such as comorbidities, use of other medications, and alcohol consumption — were not included in the analysis. Future studies could address these limitations by including more detailed information on ethnic group to better understand cultural influences on treatment decisions. They could also consider changes in patient characteristics over time and account for the use of newer medications such as SGLT2 inhibitors and GLP-1 receptor agonists.

### Comparison with existing literature

Glycaemic control directly, and diabetes-related complications indirectly, play a role in insulin initiation for individuals with T2DM.^35^ Poor glycaemic control, indicated by elevated HbA1c levels, aligns with an increased likelihood of transitioning to insulin therapy according to established clinical guidelines.^2,22,36^ Timely initiation of insulin is often indicated when oral antidiabetic agents fail to achieve adequate glycaemic control. The HbA1c target for individuals in this study cohort (ages 40–79 years) depends on age, disease duration, and treatment stage. For patients aged <70 years, the recommended target is ≤53 mmol/mol. In patients aged >70 years, the target remains ≤53 mmol/mol during the initial stage of medication, but increases to 54–58 mmol/mol for those with a disease duration of <10 years, and 54–64 mmol/mol for those with a disease duration of ≥10 years.^22^ Maintaining HbA1c below these thresholds signifies better glycaemic control and reduces complications associated with elevated blood glucose levels. As expected, an elevated HbA1c was associated with a 54% higher hazard of insulin initiation within 5 years. These findings are consistent with other studies observing elevated baseline HbA1c levels in the group that initiated insulin.^13,20,37^ This emphasises the importance of glycaemic control shortly after commencing treatment with metformin in predicting treatment trajectory and diabetes progression in individuals with T2DM.

Additionally, eGFR serves as a critical biomarker affecting insulin prescription.^38^ Lower eGFR indicates impaired kidney function, a common complication in individuals with diabetes. Providers may initiate insulin therapy earlier in individuals with compromised renal function, especially when other antidiabetic medications are contraindicated because of critically low eGFR levels, to ensure safe and effective treatment.^39^ In the current analysis, a higher eGFR was associated with a decreased probability of using insulin, in concordance with the anticipated outcome.

The observed correlation between younger current age and the risk of early insulin initiation aligns with the guidelines’ age consideration in targeted HbA1c levels and the existing literature on the natural progression of T2DM.^40^ Individuals diagnosed at a younger age, such as in their early 40s, often experience a more aggressive form of diabetes, leading to greater beta-cell dysfunction and increased insulin resistance over time.^41,42^ Additionally, challenges in changing longstanding dietary habits can exacerbate the condition. Prolonged exposure to poor dietary choices, often established early in life, may lead to persistent difficulties in glycaemic control, necessitating more intensive therapeutic interventions, such as insulin, at an earlier stage.^43^

Factors beyond glycaemic control and comorbidities can also influence insulin initiation in a primary care setting. Individuals with a migration background were less likely to receive insulin prescriptions in this study, despite research indicating that ethnic minorities, including Surinamese, have a higher prevalence of the disease, poorer glycaemic control, and an increased risk of complications.^44^ These patients also tend to experience more severe disease progression at a younger age compared with other ethnic groups, which typically contributes to a higher likelihood of earlier insulin initiation in these populations.^45,46^

The complexity of tailoring treatment plans for diverse patient populations is evident when ethnicity intersects with other variables. In diabetes management, ensuring patient adherence to insulin therapy is critical.^47^ Healthcare providers must assist patients in understanding and following insulin use instructions, as misuse poses serious risks. Such instructions require ample time for patients with limited health literacy and language barriers.^11^ The National Dutch General Practitioners Association recommends special consideration for certain patient groups, such as those with limited education, limited health literacy, and migration backgrounds, when planning insulin initiation.^22^ This approach can introduce unconscious bias when providers become hesitant to initiate insulin among such groups, even if they have elevated HbA1c levels. Clinical inertia, which refers to the delay in initiating or intensifying treatment despite established guidelines, plays a significant role in the decision-making process for initiating insulin therapy in this study.^48^ Ethnic disparity in the healthcare system is common worldwide.^49^ For instance, in high-deprivation areas such as Glasgow, South Asian patients are less likely to receive insulin compared with other ethnic groups.^50,51^ Studies in the US and UK using cross-sectional designs and randomised control trials also demonstrate a decreased likelihood of insulin prescriptions among those from ethnic minority groups.^18,20,52^ These disparities are not solely because of variations in illness duration or control, suggesting deeper cultural or physician perceptions of different ethnic groups.^53^ Insulin initiation is often delayed until significant diabetes-related complications arise, potentially exposing disadvantaged patient groups to higher risks.^17^

Furthermore, the current study found that patients with lower income were more likely to receive insulin prescriptions within 5 years after initiation of metformin. It is well established that individuals’ SES significantly influences their health and overall quality of life.^54^ For those with T2DM, lower SES is linked to higher rates of complications and mortality.^55^ This relationship is attributed to the tendency of individuals with limited education and social class achievements to require higher insulin doses and struggle with controlling their blood sugar levels, compared with peers who are better educated and with higher SES. The prevalence of obesity, smoking, and physical inactivity is higher among low-income individuals.^56^ This, combined with possible limited access to healthcare services and lower health literacy, can lead to poor glycaemic control and the need for insulin treatment earlier.^49^

The seemingly contradictory findings that individuals with migration backgrounds are less likely to receive insulin prescriptions, coupled with the observation that patients with lower SES are more likely to receive insulin treatment, raises important considerations regarding potential barriers in healthcare delivery. One of these barriers could be the language barrier, as physicians may encounter challenges in effectively communicating insulin initiation plans with patients with limited proficiency in the dominant language. Studies have shown that language barriers can hinder patient–provider communication, leading to misunderstandings about treatment recommendations and reduced adherence to therapy instructions.^57^ This issue is particularly relevant in culturally diverse healthcare settings, where patients from various ethnic backgrounds may have different linguistic preferences and levels of health literacy.^58^ Therefore, addressing language barriers through targeted interventions in future studies, such as interpreter services or culturally sensitive communication strategies, is essential to ensure equitable access to insulin therapy and optimise diabetes management outcomes.

### Implications for research and practice

This study shows that individuals with backgrounds other than Dutch, particularly those with Surinamese and Turkish backgrounds, and those with a higher income had a lower likelihood of insulin initiation, alongside established biological risk factors. The disparity in insulin prescription rates among non-native groups suggests potential unconscious biases and highlights the need to investigate barriers that may hinder patient–provider communication, such as time constraints, language differences, and limited resources. Earlier initiation of insulin in the group with low SES may reflect suboptimal diabetes management in this population, necessitating earlier medication intensification. By addressing these structural and communication barriers, healthcare systems can work towards reducing disparities and improving diabetes outcomes across diverse patient populations.

## Data

The data used in this study are restricted to researchers involved in the project, ensuring strict adherence to data integrity and confidentiality protocols. Access is limited to comply with privacy regulations and to prevent unauthorised use. Researchers interested in accessing the data for legitimate purposes may submit requests through ELAN (https://www.elanresearch.nl).
